# Supplementary material for: Prolactin at moderately increased levels confers a neuroprotective effect in non-secreting pituitary macroadenomas
Source: PLoS One. 2022 Aug 3;17(8):e0271690. doi: 10.1371/journal.pone.0271690 (PMC9348739; doi:10.1371/journal.pone.0271690)
Supplement: S1 Fig — A) Classic Mahalanobis distance is plotted for each data point of the NS+ group mean deviation with a threshold of 3. B) A QQ plot of the Mahalanobis distance and Chi-square quantile further identifies outliers with an extreme outlier at the 7th quantile corresponding to the 18th data variable in A. (DOCX) [file pone.0271690.s001.docx]

**SUPPLEMENTAL DATA:**

**Supplemental Figure 1. Outlier identification of NS+ mean deviation vs prolactin**

1. Identify Outlier

**B.**

18

**Supplemental Figure 1.** Outlier identification was determined using Mahalanobis distance. **A)** Classic Mahalanobis distance is plotted for each data point of the NS+ group mean deviation with a threshold of 3. **B)** A QQ plot of the Mahalanobis distance and Chi-square quantile further identifies outliers with an extreme outlier at the 7^th^ quantile corresponding to the 18^th^ data variable in A.
