# Supplementary material for: Prolactin at moderately increased levels confers a neuroprotective effect in non-secreting pituitary macroadenomas
Source: PLoS One. 2022 Aug 3;17(8):e0271690. doi: 10.1371/journal.pone.0271690 (PMC9348739; doi:10.1371/journal.pone.0271690)
Supplement: Attachment — Submitted filename: Response to Reviewers.docx [file pone.0271690.s002.docx]

Dear Editorial Team,

Thank you for the opportunity to revise our manuscript, *Prolactin at moderately increased levels confers a neuroprotective effect in non-secreting pituitary macroadenomas,* for publication in PLOS One. We are grateful to the reviewer for their constructive comments, which we have addressed in their entirety below. We believe the revisions have strengthened the paper. In particular, we are grateful for the reviewer’s suggestions to develop with greater clarity a discussion of the proposed mechanisms that can explain how moderate levels of prolactin demonstrate a neuroprotective effect. The changes to the paper are reflected in the “Tracked Changes” version uploaded to the publication server, with highlights and additional comments provided below. All minor reviewer changes are addressed directly within the manuscript. Deidentified supporting data have been uploaded to figshare with the following DOI: 10.6084/m9.figshare.19678953 and made available to the editors for review using the following private link: <https://figshare.com/s/af43dda8442c2f49faf4>.

**Responses to Highlighted Reviewer’s Comments:**

1. Even if several effects of PRL are now known to not depend on the sex, main ones do depend on the sex. It is therefore necessary to clearly study this issue when one deals with PRL. Biological sex is not mentioned in the Materials and Methods section. P.7 lines 171-172: the authors state “There was no difference in male/female make-up between the PRO and Control group”. Which figure does support this data? Line 171 p.7, the “male/female” terms refer to animals. Please correct

**Response:** We have included a statement acknowledging biological sex in the methods section. We also refer readers to Table 1, which includes demographic data on biological sex. Male and Female are used to describe biological sex as documented in the medical chart.

p.5 lines 130-131

Both Male and Female patients were included in the study, as defined by their biological sex on chart review.

p.8 lines 213-214

There was no significant difference in biological sex between patients in the PRO and Control groups (**Table 1)**.

2. What is the rationale to eliminate what the authors consider as “extreme outliers”? Working with confidence intervals may be a good way to solve this type of issues. In this line, I did not find any estimate of sample size to reach statistically significant conclusions. Even if the study is retrospective, such estimate is necessary. At least to get an idea of the power of the statistical data.

**Response:** Re: Outlier analysis. We have provided further context for the use of this method within the body of the paper, emphasizing how this is the most objective way to identify a potentially erroneous value. Additional post-hoc validation was also performed.

p.10 lines 251-257

In plotting the mean deviation data, a single data point varied from the rest. Prior to running statistical analysis on the data set, Mahalanobis distance was used – which identified an extreme outlier in the mean deviation data for the NS+ group (See **Supplemental Data**). This was validated post-hoc using the extreme studentized deviate test (z-score 3.66, p <0.05). Mahalanobis distance and extreme studentized deviate test did not identify any other outliers in either the PRO or NS groups. The extreme outlier was removed from the mean deviation analysis.

Re: Sample size estimate and power analysis. A power analysis was performed based on data publicly available in the literature and added to the methods section of the manuscript.

p.5 lines 140 -150

*Power analysis:* A power analysis was conducted based on the initial hypothesis that elevated levels of prolactin in prolactin-secreting macroadenoma patients will demonstrate attenuation of injury to the retinal nerve fiber layer compared with non-secreting macroadenoma patients. Previous research indicates that pre-operative mean RNFL thickness in pituitary macroadenoma patients (standard deviation) is 81.9 µm (8.8) (Moon et al.). We anticipated a similar mean RNFL thickness for non-secreting pituitary macroadenoma patients in this study. For patients with elevated prolactin, we anticipated a mean RNFL thickness to be closer to the control group mean of 91.9 µm (9.7), as reported in Moon et al. A total sample size of 30 participants (15 per group) was found to provide 80% power to detect a 10 µm difference in mean RNFL thickness between the two groups, using a Welch’s T-test and a 5% significance level.

References:

1. Moon CH, Hwang SC, Kim BT, Ohn YH, Park TK. Visual prognostic value of optical coherence tomography and photopic negative response in chiasmal compression. Invest Ophthalmol Vis Sci. 2011;52(11):8527-33.

3. p.7, line 181: What is the physiological relevance of the temporal quadrant of the retinal nerve fiber layer? Please define the superior, nasal, temporal, and inferior quadrants in the Methods section.

Response: We have included additional text that better defines the quadrants, their structural/functional relationship to the visual field, and increased sensitivity of the temporal RNFL quadrant to chiasmal compression.

p.6 lines 170-179

Measures of RNFL thickness were reported as a function of clock hour position of the fovea and subsequently grouped into anatomical quadrants based on a standard division of the visual field (e.g., for right eyes; superior quadrant: 11 and 1 o’clock; nasal quadrant: 2, 3, and 4 o’ clock; inferior quadrant: 5 and 7 o’ clock; and the temporal quadrant: 8, 9, and 10 o’clock). The 12 o’clock and 6 o’clock positions were excluded from analysis secondary to nontemporal overlap of retinal ganglion cell projections (Schneider et al.). The temporal RNFL quadrant demonstrates increased sensitivity to injury with pituitary tumors secondary to its association with crossing retinofugal fibers at the level of the optic chiasm (Moon et al.).

References:

1. Schneider CL, Prentiss EK, Busza A, Matmati K, Matmati N, Williams ZR, et al. Survival of retinal ganglion cells after damage to the occipital lobe in humans is activity dependent. Proc Biol Sci. 2019;286(1897):20182733.
2. Moon CH, Hwang SC, Kim BT, Ohn YH, Park TK. Visual prognostic value of optical coherence tomography and photopic negative response in chiasmal compression. Invest Ophthalmol Vis Sci. 2011;52(11):8527-33.

4. About the tumor characteristics, the authors state that “Tumor size was identified by a single trained investigator and the largest dimension in any direction was recorded” (p.5, lines 135-136). The tumor size should be measured. Where are they recorded?

Response: Tumor size is reported in Table 1. The text has been updated to better reflect this.

p.8 line 217-218

There was no significant difference in tumor size between the PRO (22.1 mm $\pm$ 14.7) and control groups (25.1 $\pm$ 8.9; p-value = 0.20). See **Table 1**.

5. Please detail the PRL assay (p.5 line 139).

Response: Additional text has been added to describe the PRL assay.

p.6 lines 160-166

Briefly – antigen-specific monoclonal antibodies are coated onto beads and mixed with the PRL sample to allow an immune reaction to occur. Unbound sample is then washed away and a second monoclonal antibody with an electrochemiluminescent probe is added to the mixture to bind the PRL-antibody complex. An electrode is then introduced to the sample, which generates quantifiable electrochemiluminescence via an oxidation-reduction reaction that directly correlates to the amount of PRL present.

6.  p.5-6 (lines 145 and 149): the number of tested eyes in the control group does not match. Please correct

Response: We are very grateful to the reviewer for seeing this--we identified a typo in the submitted manuscript. The total number of eyes in the prolactinoma group is 27, and for the control group 104. The manuscript text has been updated to reflect this.

7. p.6, line 158: what are normal levels of serum PRL?

Response: The normal reference range for prolactin obtained in our laboratory is 4.8-23.3ng/ml.

p.6 line 159-160.
Serum prolactin was measured via Electrochemiluminescence Immunoassay (ELISA) with a reference range of 4.8-23.3ng/ml…

8. Please discuss why a moderate increase in serum PRL levels is beneficial, while a greater increase is not, from a mechanistic point of view. In this line, p.13, lines 293-295, the authors mention that opposite actions of PRL may be due to the fact that PRL exerts both inhibitory and excitatory actions and that PRL regulates gonadotropin release. This seems to imply that the opposite actions of PRL on RFNL are due to the activation of opposite signaling pathways, which may happen, but what directs the resulting effect of PRL. Why at high doses, the neurotoxic effects would prevail and at moderate doses, the neuroprotective effects would be more important? It seems to me that this explanation does not take into account that the PRL receptors respond to their agonist with a bell-shape curve. I am referring to the fact that at high concentrations of PRL, PRL receptors desensitize. Conversely, small amount of agonist activates poorly the receptor. This may explain why the neuroprotective effect is lost if patients have too much or too little of PRL. In addition, PRL can be transformed into active peptides (vasoinhibins/16K prolactin), which also have neuronal effects. Please, complete the discussion to fully discuss this crucial issue for the study. At last, what are the actions of gonadotropin on RNFL/neuronal survival?

Response: We appreciate the reviewer’s suggestion and have bolstered the discussion section to address these key issues – particularly with respect to proposed mechanisms that may be responsible for prolactin’s effect at moderate levels. With regards to the role of gonadotropin on RNFL/neuronal survival, we felt commenting on this would be beyond the scope of the current article.

p.15 lines 370-380

Alternatively, chronic, extremely elevated levels of PRL in the PRO group can cause receptor desensitization and downregulation thus preventing the neuroprotective potential. The kinetics of the human prolactin receptor have shown it to behave with its agonist in a bell-shaped fashion implying supersaturation at high levels and decreased pathway activation at low level (Kinet et al.) Thus, the moderately elevated levels of PRL may work at peak PRL receptor activity without desensitization. Additionally, proteolytic cleavage of PRL generates a 16K PRL called vasoinhibin – which has been shown to have effects on vasculature by promoting vasopressin release, and on neurons by inhibiting neurite outgrowth (Castillo et al.). High levels of PRL would in turn result in increased levels of vasoinhibin, whose function may prevent or oppose the neuroprotective action of PRL.

References:

1. Kinet S, Bernichtein S, Kelly PA, Martial JA, Goffin V. Biological properties of human prolactin analogs depend not only on global hormone affinity, but also on the relative affinities of both receptor binding sites. J Biol Chem. 1999;274(37):26033-43.
2. Castillo X, Melo Z, Varela-Echavarria A, Tamariz E, Arona RM, Arnold E, et al. Vasoinhibin Suppresses the Neurotrophic Effects of VEGF and NGF in Newborn Rat Primary Sensory Neurons. Neuroendocrinology. 2018;106(3):221-33.

9. p. 12, lines 283-283: The authors mention that their control group is older than their PRL group. Then they discuss the implication of aging in RNFL thickness, but could they also include that PRL levels also decrease with age? It would reinforce their point that physiological PRL levels are neuroprotective and that extreme serum PRL levels, either too high or too low, are detrimental for neurons.

Response: We appreciate the reviewer’s suggestion to include this info and have added it to the discussion, along with relevant citations.

p.14 lines 351-358

Additionally, a 24-hour PRL collection study found that older patients had a lower pulse mass and lower peak values of PRL secreted (Roelfsema et al.), although average PRL values overall did not significantly decline with age. Taken together, our data demonstrates that even with the added variable of age there is a preservation of retinal thickness in the group with mildly elevated prolactin. In other words, regardless of the natural retinal thinning and PRL changes that may come with age, older patients with moderately elevated PRL had greater RNFL thickness than older patients with significant hyperprolactinemia.

References:

1. Roelfsema F, Pijl H, Keenan DM, Veldhuis JD. Prolactin secretion in healthy adults is determined by gender, age and body mass index. PLoS One. 2012;7(2):e31305.

10. p.4 lines 104-108: Please add references that support the two ways through which serum PRL can increase in humans.

Response: Two references have been added to support the mechanisms by which PRL can increase in humans.

1. Serri O, Chik CL, Ur E, Ezzat S. Diagnosis and management of hyperprolactinemia. CMAJ. 2003;169(6):575-81.
2. Mancini T, Casanueva FF, Giustina A. Hyperprolactinemia and prolactinomas. Endocrinol Metab Clin North Am. 2008;37(1):67-99, viii.

11. p.4 lines 11-112: I do not understand the logic behind the use of NS+ and NS in the control group… Does N stand for normal and S for… serum?

Response: NS stands for “non-secreting.” The text has been updated to better reflect this and to eliminate any confusion. NS+ represents “non-secreting” macroadenoma patients with an elevation of prolactin secondary to stalk effect. See p.4 lines 110-112. Thank you for this request to clarify.

12.  Table 1: The p value for the age at diagnosis needs to be checked and aligned.

Response: This value was missing a leading zero. This has been corrected. Thank you.

**Responses to Minor Reviewer’s Comments:**

p.4, line 102: “[…] visual pathway structures, we can explore […]”. A coma seems to be missing.

Corrected, thanks.

p.5, lines 126-130: The institution's reference sounds repetitive.

The institution reference has been removed.

It would be easier to detect the figures and tables in the Result section if they were written in bold.

All references to figures are now in bold type.

Please regroup all figure legends, including table legends at the end of the manuscript.

Table Legends remain with the Tables in accordance with PLOS One Formatting guidelines.

p.9 lines 203: “Post-hoc” should be written in italics.

Corrected, thanks.

p.10, line 238: Please correct typo “[…] our data demonstrate that […]”.

Corrected, Thank you.

p.11 line 260, please add a coma before but.

Done.

p.13, lines 293-294: please add a coma after hypothalamus.

Done.

**Responses to Editor’s Comments:**

Please provide additional details regarding participant consent. In the ethics statement in the Methods and online submission information, please ensure that you have specified (1) whether consent was informed and (2) what type you obtained (for instance, written or verbal, and if verbal, how it was documented and witnessed). If your study included minors, state whether you obtained consent from parents or guardians. If the need for consent was waived by the ethics committee, please include this information.

If you are reporting a retrospective study of medical records or archived samples, please ensure that you have discussed whether all data were fully anonymized before you accessed them and/or whether the IRB or ethics committee waived the requirement for informed consent. If patients provided informed written consent to have data from their medical records used in research, please include this information.

We have addressed all editorial comments in the body of the manuscript.

p.5 lines 136-138

The study protocol was approved by the institutional review board of the University of Rochester and the need for consent was waived. All data were anonymized before being accessed.

Please include captions for your Supporting Information files at the end of your manuscript, and update any in-text citations to match accordingly. Please see our Supporting Information guidelines for more information: [http://journals.plos.org/plosone/s/supporting-information](https://urldefense.proofpoint.com/v2/url?u=http-3A__journals.plos.org_plosone_s_supporting-2Dinformation&d=DwQGaQ&c=4sF48jRmVAe_CH-k9mXYXEGfSnM3bY53YSKuLUQRxhA&r=xJkUrsZmTFeTD2Ql4cLHGtx6VDTefS2WYchEBuqUrBc&m=QCdyiBwLHv-ruVFx5q6LVdnkxFGu1MVL_M0E0IbKWMA&s=fn8x6BEsblX2vchyUzpvVWj2UOJ8ayAp5xQF-rARk9o&e=).

The Caption for the Supplemental Figure has been added at the end of the manuscript.

Amended Funding Statement.

The funding statement was removed from the manuscript and is placed here per the editor’s request.

**Funding:** DP was supported by a UL1 TR002001 grant via the NIH/National Center for Advancing Translational Sciences at the University of Rochester and resources provided by the Program for Translational Brain Mapping at the University of Rochester. The program for Translational Brain Mapping was established, in part, with support from Norman and Arlene Leenhouts (see: www.tbm.urmc.rochester.edu). AR was supported by a CTSI TL1 TR000096 grant via the National Center for Advancing Translational Sciences at the University of Rochester.

Thank you again for the opportunity to improve upon our manuscript.

Sincerely,

David A. Paul, MD, MS
(On behalf of all authors)
