# Supplementary material for: Prolactin at moderately increased levels confers a neuroprotective effect in non-secreting pituitary macroadenomas
Source: PLoS One. 2022 Aug 3;17(8):e0271690. doi: 10.1371/journal.pone.0271690 (PMC9348739; doi:10.1371/journal.pone.0271690)
Supplement: Attachment — Submitted filename: Response to Reviewers.docx [file pone.0271690.s003.docx]

Dear Editorial Team,

Thank you for the opportunity to revise our manuscript, *Prolactin at moderately increased levels confers a neuroprotective effect in non-secreting pituitary macroadenomas,* for consideration at PLOS One. All reviewer comments have been addressed as outlined below.

**Responses to Reviewer’s Comments:**

1. Please specify the antibody (manufacturer, ref.) used for the PRL assay.

Additional Editor Comments (if provided): Please provide the additional information requested by the reviewer. In particular, please describe not just the source of the antibody used, but the evidence demonstrating it specificity (western blot analysis, immunostaining of transfected cells, analysis in KO mice, etc.). If that evidence is derived from other published papers, please provide the references to them.

**Response:** The manuscript has been updated to include the PRL assay manufacturer (Roche Diagnostics; Indianapolis, IN) and evidence of its sensitivity to detect prolactin (see new reference below, [16]). Of note, we have also included text to clarify that PRL testing was conducted in a CLIA certified clinical laboratory as part of the routine clinical workup for pituitary tumor patients at our institution.

16.	Fahie-Wilson M, Bieglmayer C, Kratzsch J, Nusbaumer C, Roth HJ, Zaninotto M, et al. Roche Elecsys Prolactin II assay: reactivity with macroprolactin compared with eight commercial assays for prolactin and determination of monomeric prolactin by precipitation with polyethylene glycol. Clin Lab. 2007;53(5-6):301-7.

*Page 6, lines 149-156*

*Measurement of Serum* *Prolactin Levels*: Serum prolactin was measured as part of the routine clinical workup for each patient via FDA approved Roche Elecsys Prolactin II Assay (Electrochemiluminescence Immunoassay [ECLIA]; Roche Diagnostics; Indianapolis, IN) with a reference range of 4.8-23.3ng/ml [15]. This assay demonstrates increased sensitivity to detect the concentration of monomeric prolactin by avoiding false elevation secondary to reactivity with macroprolactin [16]. Our clinical laboratory is a Clinical Laboratory Improvement Amendments (CLIA) certified laboratory and accredited by the Colleague of American Pathology and New York State Department of Health.

2. In the discussion about the potential mechanistic explanation for the opposite roles of PRL according to its levels, the "vasoinhibin" term should be used in plural. This was the whole point renaming them vasoinhibins instead of 16K prolatin, which infers that only 16 kDa N-terminal fragments are produced.

**Response:** The text has been updated per the reviewers’ comments.

*Page 15, lines 346-350*

Additionally, proteolytic cleavage of PRL generates active peptides (vasoinhibins/16K PRL) – which have been shown to have effects on vasculature by promoting vasopressin release, and on neurons by inhibiting neurite outgrowth [32]. High levels of PRL would in turn result in increased levels of vasoinhibins, whose function may prevent or oppose the neuroprotective action of PRL.

**Additional minor edits:**

1. The acknowledgment section was updated to include Victoria Zhang, PhD for her assistance with the clinical laboratory protocols.

*Page 16, lines 366-368.* The authors are grateful to Peggy Auinger, MS for her contribution to the statistical analyses and for her comments on earlier drafts of the paper, and to Victoria Zhang, PhD for her assistance with the clinical laboratory protocols used in the evaluation of PRL.

2. Lastly, the word, “older” on pg. 14, line 239 was removed during the editing process. The second use of the word in this sentence was found to be a typo and thus deleted for clarity.

Thank you again for the opportunity to improve upon our manuscript.

Sincerely,

David A. Paul, MD, MS
(On behalf of all authors)
